# Uncovering hidden sensorimotor memories in mice with Alzheimer’s disease relevant pathology

**DOI:** 10.1002/alz.089282

**Published:** 2025-01-03

**Authors:** Andrea Santi, Sharlen Moore, Aaron Wang, Jennifer Lawlor, Kelly Fogelson, Kishore Kuchibhotla

**Affiliations:** ^1^ Johns Hopkins University, Baltimore, MD USA; ^2^ University of California San Diego, San Diego, CA USA

## Abstract

**Background:**

Alzheimer’s disease is a progressive form of dementia where cognitive capacities deteriorate due to neurodegeneration. Interestingly, Alzheimer’s patients exhibit cognitive fluctuations during all stages of the disease. Though it is thought that contextual factors are critical for unlocking these hidden memories, understanding the neural basis of cognitive fluctuations has been hampered due to the lack of behavioral approaches to dissociate memories from contextual‐performance. Our previous work demonstrated that interleaving reinforced with non‐reinforced (‘probe’) trials in an auditory go/no‐go discrimination task, allows us to distinguish between acquired sensorimotor memories and their contextual expression.

**Method:**

Here, we used this approach, together with two‐photon calcium imaging on behaving APP/PS1+ mice, to determine whether amyloid accumulation impacts underlying sensorimotor memories and/or contextual performance in an age dependent manner.

**Result:**

Importantly, peripheral auditory function, measured using auditory brainstem responses, was similar between WT and APP/PS1+ mice. We found that while contextual‐performance is significantly impaired in 6‐8mo APP/PS1+ mice compared to age‐matched controls, these animals have preserved sensorimotor memories. However, 12mo APP/PS1+ mice show deficits in both domains. The impairment found in the young adults was accompanied by overall network suppression, compensatory disinhibition, reduced stimulus selectivity, and aberrant behavioral encoding in neurons of the primary auditory cortex that was partially restored in probe trials. These impairments were concentrated near Aβ plaques and were recapitulated with a reinforcement learning model that accounts for contextual signal changes. Model parameters affected were those governing contextual scaling and behavioral inhibition.

**Conclusion:**

These results suggest that Aβ deposition impacts circuits involved in contextual computations before those involved in acquiring knowledge and that neural circuit interventions (modulating inhibition) may hold promise to reveal hidden memories.

